# P01.31. Yoga therapy associated with increased brain GABA levels and decreased depressive symptoms in subjects with major depressive disorder: a pilot study

**DOI:** 10.1186/1472-6882-12-S1-P31

**Published:** 2012-06-12

**Authors:** C Streeter, P Gerbarg, R Saper

**Affiliations:** 1 Boston University School of Medicine, Boston, USA; 2 New York Medical College, Kingston, USA

## Purpose

It is hypothesized that imbalance of the autonomic nervous system (ANS) with decreased parasympathetic nervous system (PNS) activity and underactivity of the gamma amino-butyric acid (GABA) system contribute to symptoms in Major Depressive Disorder (MDD) and that yoga-based practices correct underactivity of the PNS and GABA systems which results in decreased depressive symptoms.

## Methods

Normal subjects (n=19) and those with MDD (n=2) participated in two comparable 12-week manualized yoga asana interventions. Both groups had thalamic GABA levels measured by magnetic resonance spectroscopy before (Scan 1) and after the 12-week intervention (Scan 2). Depressive symptoms were measured using the Physicians Health Questionnaire (PHQ)-9 prior to each scan.

## Results

MDD subjects (n=2) had PHQ-9 scores of 22 (severe depression) and 20 (moderately severe depression) at the beginning of the yoga intervention and scores of 7 (mild depression) and 4 (not depressed enough to be considered MDD) at the end of the study, respectively. The MDD subjects had mean thalamic GABA/Creatine ratios (GABA Levels) of 0.039 ± 0.004 for Scan 1 and mean GABA levels of 0.049 ± 0.010 for Scan 2 for a change of 0.014 ± 0.006. Normal subjects had mean thalamic GABA levels of 0.065 ± 0.021 for Scan 1, and 0.061 ± 0.021 for Scan 2, for a change of -0.004± 0.017.

## Conclusion

The results from this small pilot study are consistent with the proposed theory that predicts 1) the lower GABA levels found in subjects with MDD, 2) MDD GABA levels increased towards those of normal subjects after the yoga intervention, 3) improved mood was associated with increased GABA levels, 4) subjects remained symptomatic with low GABA levels despite pharmacologic treatment until they received the yoga intervention that presumably corrected their PNS imbalance, after which GABA levels increased and depressive symptoms decreased.

